# Machine learning assisted dynamic phenotypes and genomic variants help understand the ecotype divergence in rapeseed

**DOI:** 10.3389/fpls.2022.1028779

**Published:** 2022-11-15

**Authors:** Hui Feng, Chaocheng Guo, Zongyi Li, Yuan Gao, Qinghua Zhang, Zedong Geng, Jing Wang, Guoxing Chen, Kede Liu, Haitao Li, Wanneng Yang

**Affiliations:** ^1^ National Key Laboratory of Crop Genetic Improvement, National Center of Plant Gene Research (Wuhan), Hubei Hongshan Laboratory, Huazhong Agricultural University, Wuhan, China; ^2^ State Key Laboratory of Biocatalysis and Enzyme Engineering, and Hubei Collaborative Innovation Center for Green Transformation of Bio-resources, School of Life Sciences, Hubei University, Wuhan, China

**Keywords:** machine learning, dynamic phenotyping, quantitative trait loci, ecotype, rapeseed

## Abstract

Three ecotypes of rapeseed, winter, spring, and semi-winter, have been formed to enable the plant to adapt to different geographic areas. Although several major loci had been found to contribute to the flowering divergence, the genomic footprints and associated dynamic plant architecture in the vegetative growth stage underlying the ecotype divergence remain largely unknown in rapeseed. Here, a set of 41 dynamic i-traits and 30 growth-related traits were obtained by high-throughput phenotyping of 171 diverse rapeseed accessions. Large phenotypic variation and high broad-sense heritability were observed for these i-traits across all developmental stages. Of these, 19 i-traits were identified to contribute to the divergence of three ecotypes using random forest model of machine learning approach, and could serve as biomarkers to predict the ecotype. Furthermore, we analyzed genomic variations of the population, QTL information of all dynamic i-traits, and genomic basis of the ecotype differentiation. It was found that 213, 237, and 184 QTLs responsible for the differentiated i-traits overlapped with the signals of ecotype divergence between winter and spring, winter and semi-winter, and spring and semi-winter, respectively. Of which, there were four common divergent regions between winter and spring/semi-winter and the strongest divergent regions between spring and semi-winter were found to overlap with the dynamic QTLs responsible for the differentiated i-traits at multiple growth stages. Our study provides important insights into the divergence of plant architecture in the vegetative growth stage among the three ecotypes, which was contributed to by the genetic differentiation, and might contribute to environmental adaption and yield improvement.

## Introduction

Crop species undergo multi−staged domestication from a particular center and then expand to a wider geographical distribution (Meyer and Purugganan, 2013). Long-term domestication and improvement reshape crops to diverse subspecies or ecotypes, with many geographically agronomic traits, to adapt to different agro-ecological and cultural environments. Recently, genomic changes underlying the differentiated traits among different subspecies or ecotypes have been successfully identified by high-throughput genotyping assisted population genetic analysis in many crops, which include rice (Wang et al., 2018b; Zhang et al., 2019), maize (Hufford et al., 2012; Liu et al., 2015), soybean (Zhou et al., 2015), cucumber (Qi et al., 2013), tomato (Lin et al., 2014) and cotton (Wang et al., 2017; He et al., 2021). Numerous loci/genes associated with root microbiota, plant architecture, grain yield, stress responses, and flowering time were identified and will accelerate the breeding process of new cultivars. Meanwhile, such genomic features and differentiated traits can be used as biomarkers to distinguish ecotype or subspecies (Zhang et al., 2019).

The allotetraploid rapeseed (*B. napus*) is a relatively new species and originates from interspecific hybridization between the ancestor of European turnip (*B. rapa*, A subgenome) and the common ancestor of kohlrabi, cauliflower, broccoli, and Chinese kale (*B. oleracea*, C subgenome) less than 7500 years ago (Chalhoub et al., 2014; Lu et al., 2019; Song et al., 2020). To date, the rapeseed includes three ecotypes for adapting to different geographic areas (Qian et al., 2006; Snowdon et al., 2007; Yin et al., 2020). Winter (W) ecotype is mainly in Europe, and is generally sown in autumn and flowers in late spring after a strong vernalization. Spring (S) ecotype is mainly in Northern Europe, Canada, Australia, and north-western China; it is not winter-hardy and is generally sown in spring and flowers without vernalization. Semi-winter (SW) ecotype is mainly found in the Yangtze River basin of China, and is generally sown in autumn and flowers in early spring after moderate vernalization. It has been suggested that the winter ecotype was the original form of rapeseed, and the spring and semi-winter ecotype were developed ~416 and ~60 years ago, respectively (Lu et al., 2019). Recently, millions of genomic variations among W, S, and SW ecotypes of rapeseed were identified by SNP array and whole-genome resequencing (Wei et al., 2017; Wang et al., 2018a; Lu et al., 2019; Wu et al., 2019). A panel of genes, such as *BnaA10.FLC*, *BnaA02.FLC*, *BnaA03.FLC.a BnaA02.FT*, and *BnaA03.FRI*, were identified to be associated with the flowering time divergence of three rapeseed ecotypes, by integration of selective sweep analysis, genome-wide association study (GWAS), single gene haplotype analysis, and transgenic validation (Wei et al., 2017; Yi et al., 2018; Wu et al., 2019; Yin et al., 2020). The haplotypes in these genes and flowering time could be used to distinguish the ecotype and improve the cultivar in rapeseed. However, the divergence of genomic footprints and associated dynamic plant architecture in vegetative growth stages has not yet been analyzed systematically.

Machine learning (ML) is a set of computational approaches to find predictive patterns in data and has been widely used to identify biomarkers for subspecies discrimination and yield heterosis prediction (Zhang et al., 2019; Dan et al., 2021; van Dijk et al., 2021). Previously, we developed an automatic image analysis pipeline to quantify dynamic plant architecture throughout multiple development stages in rapeseed (Li et al., 2020). In this study, this pipeline was used to study the dynamic architecture of plant growth and growth rate within a rapeseed population, including 14 W types, 24 S types, and 133 SW types. We identified that a subset of 19 i-traits contribute to divergence of the three ecotypes using the random forest (RF) model of ML. And, we analyzed genomic variations of the population, genomic basis of the ecotype differentiation, and QTL information of all dynamic i-traits. It was found that 213, 237, and 184 QTLs of differentiated i-traits overlapped with the 37, 33, and 42 ecotype differentiation regions between W and S, W and SW, and S and SW, respectively. This suggests that our identified differentiation of i-traits among the ecotypes was contributed to by the genetic variations and differentiation.

## Materials and methods

### Sample collection and phenotyping

A total of 171 rapeseed cultivars or inbred lines were collected from ten countries or regions across the world (**Supplementary Table S1**). These lines were selected from our previously published population to represent the three ecotypes of W, S, and SW (Wang et al., 2018a). Phenotyping was performed in the high-throughput rice phenotyping facility (HRPF), located in Huazhong Agricultural University, Wuhan, China (Yang et al., 2014). All rapeseed lines were sowed in the pot and screened from the seedling to the initial flowering stage at 11 time points of T1 to T11, with intervals of one week (**Supplementary Table S2**). Experimental layout and management were the same as that described previously (Li et al., 2020). The trials were performed using a randomized block design with three replications in the winter-spring growing season of 2014-2015. In total, we generated a total of 215.42 Gb of RGB images (44,118 images; PNG format), which are available in a database (http://plantphenomics.hzau.edu.cn/usercrop/Rape/image/2014-2015-GWAS, selecting of “2014-2015-GWAS”). A set of 41 dynamic i-traits (18 i-traits in top view and 23 i-traits in side view) and 30 growth-related traits reflecting the growth speed were obtained using the image analysis pipeline, described previously (**Supplementary Table S3**) (Li et al., 2020). The detailed instructions of the image analysis pipeline and the source code of programs built in the LabVIEW 2015 (National Instruments, US) can be obtained in our previous study (Li et al., 2020). Outliers were removed by “3σ” criterion; the remaining i-traits were used for subsequent phenotypic analysis.

### Identification of i-traits contributing to the divergence of ecotype

Five machine learning models, namely discriminant analysis (DCA), random forest (RF), support vector machine (SVM), multilayer perceptron (MLP), and convolutional neural network (CNN), were performed to distinguish different ecotypes, based on the 18 i-traits in top view from T1 to T11 and 23 i-traits in side view from T7 to T11, respectively. DCA was performed by IBM SPSS 20.0. RF, SVM, MLP, and CNN were built by Python3.6. The detailed parameters were as follows: (1) Bayesian basis DCA expression was established to distinguish different ecotypes and i-traits with high contribution which were acquired with stepwise establishment of discriminant expressions. (2) CART algorithm was used to split nodes in building RF. (3) SVM with radial basis kernel was built for a best decision edge with the highest confidence between ecotypes, which was searched for by adjusting the slack variable and penalty factor C. (4) Multilayer perceptron (MLP) and convolutional neural network (CNN) were activated by softmax on the last layer. For each model, the i-traits were divided into training sets and testing sets and the capability of the model was evaluated by the performance using the test set. Finally, the i-traits excavated by RF model with high contribution to divergence of ecotype were used for subsequent analysis.

### Redraw of rapeseed images from i-traits

Based on the i-traits excavated by RF model with high contribution to divergence, a text-to-images model StackGANv1 (https://github.com//hanzhanggit//StackGAN) was used to construct the rapeseed images. StackGANv1 was built by python 2.7 with tensorflow 0.12 and accelerated with GEFORCE RTX2080 SUPER. Stage I (i-traits to images) and stage II (images to images) were trained 800 times and 200 times, respectively. After the simulated images were obtained by the StackGANv1, three parameters, MSE, SSIM, and PSNR, were calculated to evaluate the image similarity between real and simulated images.

### Mapping, variant calling and annotation

Sequence data (PE100 reads) of the 171 rapeseed lines were obtained from our previous study (Wang et al., 2018a). The variants were identified again based on the newly published ZS11 genome (Song et al., 2020). Putative single nucleotide polymorphisms (SNPs) were obtained using the Burrows-Wheeler Alignment tool (BWA), SAMtools, and Genome Analysis Toolkit (GATK), according to the variant calling process that was described previously (Tang et al., 2021). The raw variants were further filtered using the following criteria: (1) the relative heterozygosity (HR) had to be less than 0.2 (Wu et al., 2016); (2) the percentage of missing genotype had to be less than 60% in the population; and (3) the confidence score from GATK had to be greater than 20. Finally, missing genotypes of all variants were imputed using Beagle software and variants with allele frequencies lower than 5% in the population were discarded. The identified SNPs were annotated using the ANNOVAR package (Wang et al., 2010), based on the ZS11 genome and annotation model (http://cbi.hzau.edu.cn/bnapus/index.php).

### Population genetics analysis

To build a phylogenetic tree and perform principal component analysis, a subset of 131,319 SNPs was selected randomly with a step of 5-kb window across the genome. These SNPs were distributed evenly among the genome and better reflect population structure and demography. The phylogenetic tree was constructed using MEGA X software (Kumar et al., 2018), with the neighbor joining method and 1000 bootstrap replicates. Principal component analysis was performed using the smartpca program in EIGENSOFT software (Price et al., 2006); with that the first two eigenvectors were used. Linkage disequilibrium (LD) between each pair of SNPs was calculated for 1,000-kb windows using PopLDdecay software (Zhang et al., 2018). The LD decay was calculated on the basis of the *r^2^* value between two SNPs in each window and plotted using custom R script.

### Ecotype differentiation analysis

Nucleotide diversity (*π*), measuring the degree of variability within a population, was calculated for 100-kb sliding windows with a step size of 10 kb using the VCFtools (Danecek et al., 2011). Population fixation statistics (*F_ST_*) was estimated for 100-kb sliding windows with a step size of 10 kb using the PopGenome (https://popgenome.weebly.com/). The average *F_ST_* from all sliding windows was used to reflect the degree of population divergence among different ecotypes. Sliding windows with the top 1% *F_ST_* value were selected as putative significant differentiated windows. Of these, the top 1% had *F_ST_* ≥0.72, 0.66, and 0.5 for W vs S, W vs SW, and S vs SW, respectively. Neighboring windows, the distance <20 kb, were then merged into one region. These regions were regarded as highly diverged across ecotype.

### Genome-wide association study

Genome-wide association study (GWAS) was performed using a mixed linear model (MLM) in genome-wide efficient mixed model association (GEMMA) software, such as that described previously (Wang et al., 2018a). The effective number of independent markers (N) was calculated using GEC tool (Li et al., 2012), and suggestive *P* value (1/N=8.67×10^-7^) was set as the significance threshold. The GWAS signals of all traits was identified according to the following two steps. Firstly, all *P* value of SNPs responsible for each i-trait were used as a query of function “clump” of PLINK software. The function was used to obtain the independent peak SNPs of each i-trait with a sliding window based on the decay of LD. The parameter of PLINK was –clump-p1 8.67×10^-7^ –clump-p2 1×10^-5^ –clump-r^2^ 0.3 –clump-kb 500 –clump-allow-overlap. The QTL intervals for each i-trait were defined as the minimum and the maximum position of the SNPs meeting these criteria. Secondly, all QTLs with overlapping intervals were categorized as nonredundant QTLs.

## Results and discussion

### Phenotypic variation

**Figure 1:**
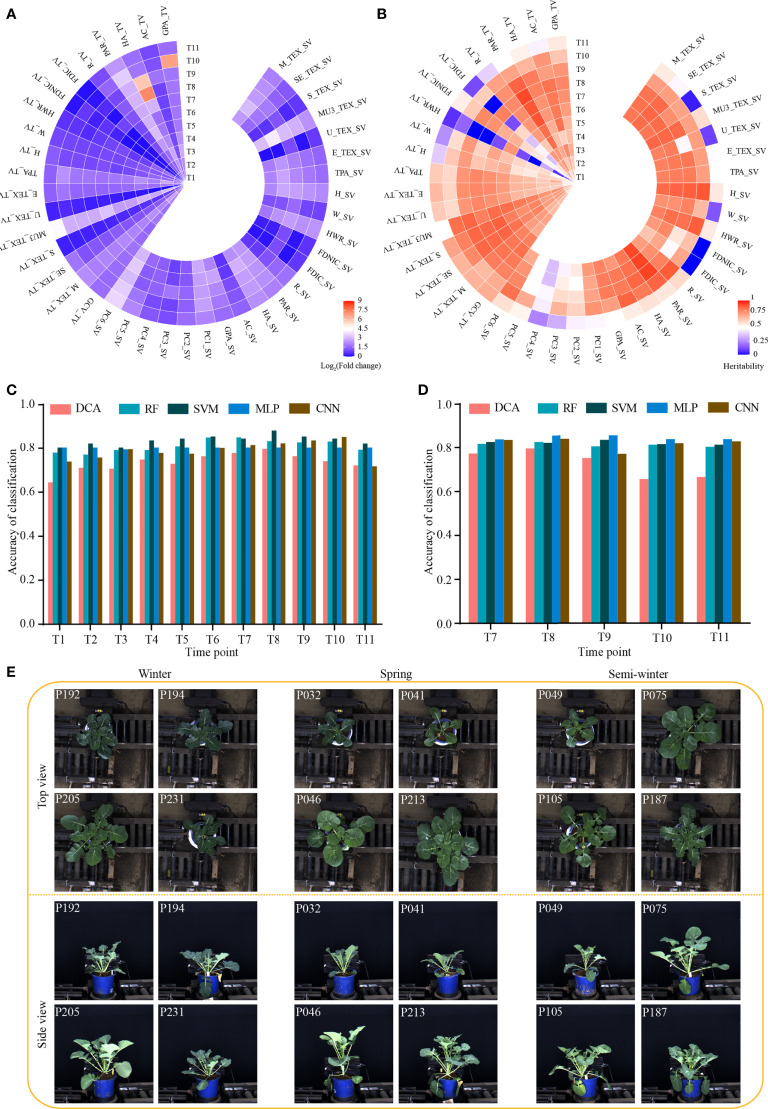
Dynamic phenotype of 43 i-traits across 11 time points and performance of models distinguishing the rapeseed ecotype. **(A)** Heat map showing the phenotypic fold change. **(B)** Heat map showing the broad-sense heritability (*H* ^2^) of traits. **(C**, **D)** Classification accuracy of five machine learning models based on the i-traits in top-view from T1-T11 **(C)** and side view from T7-T11 **(D)**. DCA, discriminant analysis; RF, random forest; SVM, support vector machine; MLP, multilayer perceptron; CNN, convolutional neural network. **(E)** Images of three rapeseed ecotypes from T7 in top view and side view. The top-left numbers indicated the No. of displayed accessions.

We evaluated the phenotypic diversity and broad-sense heritability (*H^2^*) of the 41 dynamic i-traits. The magnitude of diversity varied drastically among different i-traits in the rapeseed accessions at each time point (**Figure 1A**; **Supplementary Table S4**). The average fold change for all i-traits was 4.44, ranging from 1.04 to 147.68 among the different time points. Of which, MU3_TEX_SV/TV (Third moment of whole plant in top/side view, reflecting the complex degree of leaves) and PC6_SV (Plant compactness of whole plant traits in side view, reflecting the compactness of the whole plant) had the highest range of phenotypic variation across all time points expect time point T8-T10, with the fold change of the traits ranging from 5.16 to 19.73 (**Figure 1A**). This result was similar to that reported previously (Li et al., 2020). However, the fold change of all 41 i-traits in this study was higher than that observed in rapeseed intervarietal substitution line (ISL) population previously (Li et al., 2020). This suggests that the dynamic i-traits have a wider diversity in the nature rapeseed germplasm compared with the artificially constructed population. Among the 11 time points, the average of *H^2^* of all 41 i-traits was 0.67 (**Figure 1B**; **Supplementary Table S4**). Of these, most (39, 95.12%) i-traits have higher heritability (>0.50) at more than half of the time points. And, the i-traits generally showed the highest heritability at the time points T7 or T8 (the bud stage, **Figure 1B**). This phenomenon was also observed in the ISL population previously (Li et al., 2020).

### Performance of classification models

To identify the i-traits contributing to the divergence of three ecotypes in rapeseed, five machine learning models, namely discriminant analysis (DCA), random forest (RF), support vector machine (SVM), multilayer perceptron (MLP), and convolutional neural network (CNN), were established using i-traits in top view from T1 to T11 and side view from T7 to T11, respectively. The classification accuracy of different models through all dynamic time points were shown in **Figures 1C, D**. A high classification accuracy with similar dynamic change among the time points was observed for all five models. The average of classification accuracy ranged from 0.75 to 0.83, and 0.79 to 0.83 for i-traits in top view from T1 to T11 and side view from T7 to T11, respectively. (**Figures 1C**, **D**). Moreover, the average of classification accuracy increased gradually and reached the maximum in T7 and T8 for i-traits in both top view (0.82 and 0.83) and side view (0.82 and 0.83), although the visual difference was not particularly obvious among some accessions in the three rapeseed ecotypes, from both top and side view at T7 period (**Figure 1E**). These results suggest that the machine learning is effective in distinguishing the rapeseed ecotypes based on the dynamic i-traits.

### I-traits contributing to the ecotype divergence

**Figure 2:**
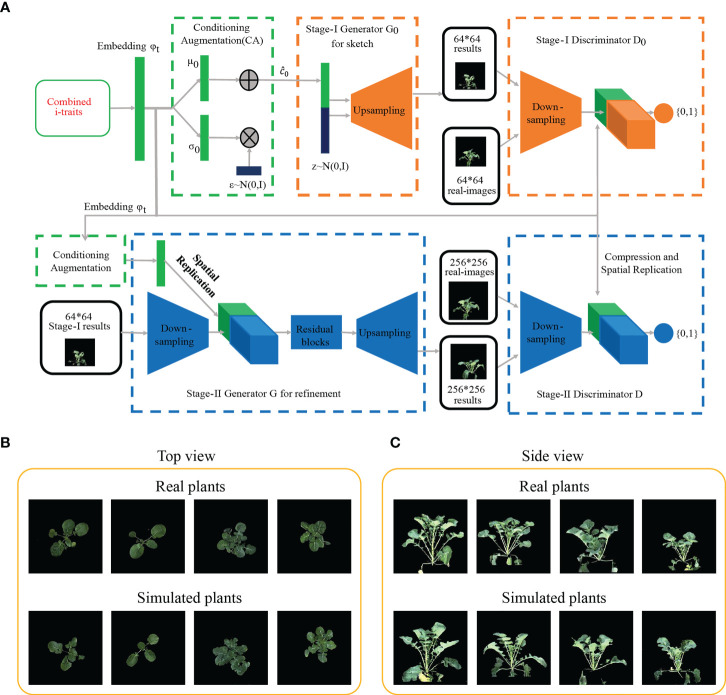
Generation of images based on machine learning. **(A)** Procedure of generating rapeseed images by the StackGANv1 model. **(B**, **C)** Comparison of images between real and simulated images generated based on the nine i-traits in top view **(B)** and ten i-traits in side view **(C)** from the top 50% i-traits screened by the RF model.

It would be beneficial if the ecotype could be distinguished in early growth stage. Notably, RF with the contributed i-traits was established and displayed an impressive accuracy at the first inspection stage of top-view (T1) and side-view (T7); 78.0% samples were correctly classified in T1 and 81.7% samples were correctly classified in T7 (**Figures 1C**, **D**). A subset of nine i-traits in top view and 10 i-traits in side view from the top 50% of i-traits were screened by RF, which contributed to the divergence of ecotype in rapeseed (**Supplementary Table S5**). To further verify whether these extracted i-traits can well distinguish the individual plants, the nine i-traits and 10 i-traits in top-view and side view were taken to train stackGAN to generate images, respectively. Model with i-traits in T1 with 4,872 images in top-view was trained for 15 hours, and model with i-traits in T7 with 22,691 images in side-view was trained for 48 hours (**Figure 2A**). Examples of coupled real and simulated images are shown in **Figures 2B, C**. It was showed that the simulated images of plant were clear and bright with smooth texture details and highly similar with the coupled real ones. Moreover, three evaluation parameters of image similarity, mean square error (MSE), structural similarity (SSIM), and peak signal-to-noise ratio (PSNR), were 0.02, 0.75, and 17.13, respectively. This observation indicates a high similarity between the simulated and real images. Taken together, these results suggest that the selected traits could represent the overall plant well and be used as biomarkers to distinguish the ecotypes.

**Figure 3:**
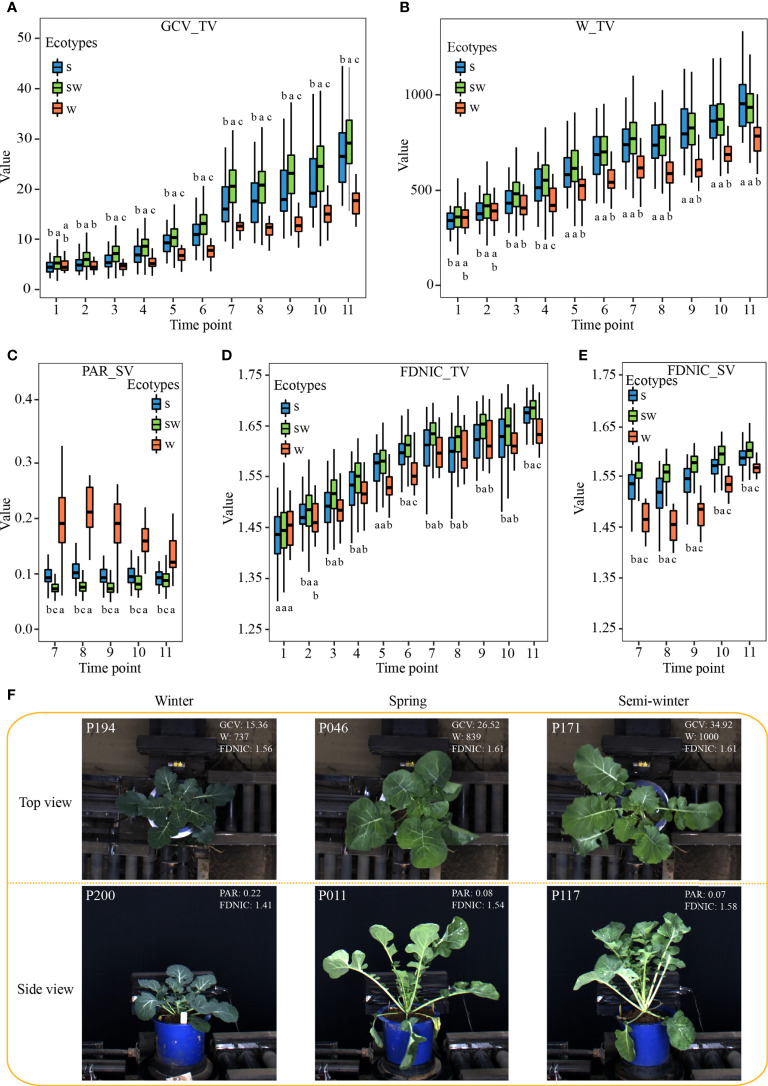
Characteristics of the subset of i-traits contributing to ecotype divergence among the three ecotypes. **(A–E)** The box plots of GCV_TV **(A)**, W_TV **(B)**, PAR_SV **(C)**, FDNIC_TV **(D)**, and FDNIC_SV **(E)** from T1 to T11 among the three rapeseed ecotypes. Differences between the ecotypes were analysed by Wilcoxon rank-sum test and different letters represent significant difference (P < 0.05). **(F)**: Plant morphology of the three rapeseed ecotypes from T7 in top view and side view. The top-left numbers indicated the No. of displayed accessions.

The plant architecture reflected by the contributed i-traits might be associated with the environmental adaption and yield improvement among the ecotypes, which are the two important breeding processes in rapeseed (Hu et al., 2022). Of these, GCV_TV (Green color value in top view) shows the green component that to some extent is negatively associated with the chlorophyll content of plants (Wu et al., 2018). There was a significant difference for the GCV_TV of each stage among the three ecotypes (**Figures 3A**, **F**). This result suggests that GCV_TV had been selected artificially during the breeding, and the W ecotype, the original form of rapeseed, had more chlorophyll content in leaf, which putatively increased photosynthesis rate and sugar content. The W_TV (Plant width in top view) and PAR_SV (perimeter/projected area ratio of whole plant in side view) are associated with the leaf angle and shape, petiole length, and plant compactness. A significant difference was observed among the three ecotypes (**Figures 3B**, **C**), which suggests that the W ecotype displayed more horizontal leaves with short petiole (**Figure 3F**). These performances may help rapeseed with overwintering during strong vernalization (Hu et al., 2022). The FDNIC_TV/SV (Fractal dimension without image cropping of whole plant in top/side view) are positively associated with the biomass and yield in rapeseed (Li et al., 2020). SW ecotype had a significantly higher value for these two i-traits of each stage than that of W and S ecotype (**Figures 3D**, **E**). This observation might suggest that the yield of modern SW accessions had been improved during the rapeseed breeding (Hu et al., 2022). In addition, similar significant differences were observed for other contributed plant morphological i-traits among the three ecotypes (**Supplementary Figures S1**, **S2**). Furthermore, SE_TEX_TV/SV (standard error of whole plant in top/side view), S_TEX_TV/SV (smoothness of whole plant in top/side view), M_TEX_TV (mean value of whole plant in top view), and MU3_TEX_TV/SV (third moment of whole plant in top/side view), which are texture traits and reflect the complex degree of leaf grayscale in vegetable state, displayed significant differences among the three ecotypes across each stage (**Supplementary Figures S1**, **S2**). This result suggests that the leaves become more complex in S and SW ecotype, compared with W ecotype, during the breeding of rapeseed.

### Genomic variation and population structure

**Figure 4:**
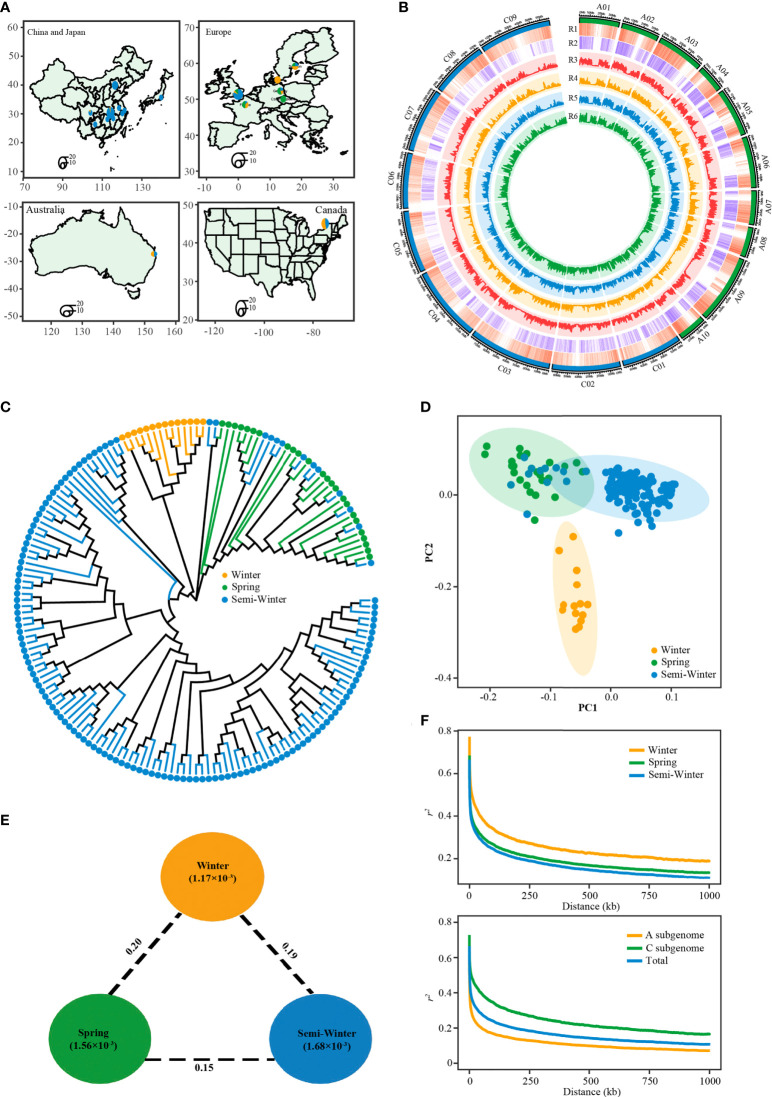
Features of the rapeseed population consisted of winter, spring and semi-winter ecotypes. **(A)** The geographic distribution of the 171 accessions. Accessions from China are represented by a circle on the provincial capital city. Accessions from other countries are represented by a circle on the capital city. Circle size indicates number of accessions. **(B)** Circos plot showing genetic diversity among three ecotypes. R1: gene density, R2: SNP density, R3: genetic diversity (*π*) in whole population, R4: genetic diversity (*π*) in winter ecotype group, R5: genetic diversity (*π*) in semi-winter ecotype group, R6: genetic diversity (*π*) in spring ecotype group. **(C)** Phylogenetic tree of all accessions inferred from a subset of 131,319 SNPs, distributed randomly across whole-genome. The yellow, green, and blue dots indicate winter, spring, and semi-winter accessions. **(D)** PCA plots of the first two components of 171 accessions. **(E)** Summary of nucleotide diversity and population divergence across the three ecotype groups. Values in circles represent measures of nucleotide diversity for the group, and values between pairs indicate ecotype divergence (*F_ST_*). **(F)** Decay of LD (*r^2^*) in the three groups (top) and two subgenomes (bottom).

The 171 rapeseed accessions consisted of 14 W ecotypes, 24 S ecotypes, and 133 SW ecotypes. These lines had a wide geographic distribution, including Europe (France, Sweden, Denmark, Germany, Czech Republic), Canada, Australia, China, and Japan (**Figure 4A**; **Supplementary Table S1**). A total of 6.83 billion paired-end reads (1.36 Tb of sequence) were obtained, with an average depth of 5.28× of the reference genome ZS11 (**Supplementary Table S1**). After mapping against the newly published genome of ZS11 (Song et al., 2020) and variants filtering, we identified a total of 5,324,005 SNPs (**Figure 4B**; **Supplementary Table S6**). Of these, 2,589,260 (48.6%), 1,166,139 (21.9%), and 1,568,606 (29.5%) were located in intergenic regions, upstream/downstream regions, and the gene body, respectively (**Supplementary Table S7**). We also identified 316,612 nonsynonymous SNPs, which caused start codon changes, gain of premature stop codons, or the production of elongated transcripts (**Supplementary Table S7**). The number and density of SNPs in the A subgenome (2,692,034; 7.08 SNPs/kb) was higher than that in the C subgenome (2,631,971; 4.61 SNPs/kb) (**Figure 4B**; **Supplementary Table S7**).

To get the overall genetic relationship among the three ecotypes in this population, we explored the phylogenetic relationship and performed principal component analysis (PCA) of 171 accessions using randomly selected SNP markers. The neighbor-joining tree revealed the accession within W, S, and SW ecotype clustered each other, (**Figure 4C**). However, there were thirteen SW accessions mixed with the S ecotype clade. This result was supported by the PCA, in which principal component PC2 separated the W ecotype from the S and SW ecotype, and PC1 separated the S ecotype from most SW ecotypes except the abovementioned mixed thirteen SW accessions (**Figure 4D**). It was found that genetic diversity of SW ecotype types (π=1.68×10^-3^) was higher than that of S (π=1.56×10^-3^) and W (π=1.17×10^-3^) (**Figures 4B**, **E**; **Supplementary Table S6**). The diversity level in all three ecotypes was similar to that reported in the larger germplasm accessions previously (Wu et al., 2019; Tang et al., 2021; Hu et al., 2022), which suggests that our population could represent genetic diversity of the three ecotypes. And, the genetic diversity in A sub-genome was higher than that in C sub-genome in all three ecotypes, with the maximum difference observed in SW ecotype (**Figure 4B**). This observation was consistent with the fact that the diversity of *B. rapa* contribute more to A genome of *B. napus* than *B. oleracea* to C genome of *B. napus* diversity (Qian et al., 2006; Sun et al., 2017). The decay of linkage disequilibrium (LD) with physical distance between SNPs (1/2 max *r^2^*) occurred at 20.6 kb in our population, with 52 kb in W ecotype, 23.5 kb in S ecotype, and 18.4 kb in SW ecotype (**Figure 4F**; **Supplementary Table S6**). The LD extent of C sub-genome was much higher than that of A sub-genome, independent of the ecotype (**Figure 4F**; **Supplementary Figure S3**). The overall LD extent and its sub-genomic pattern in our study was similar to that reported previously (Lu et al., 2019; Wu et al., 2019; Tang et al., 2021).

### QTL identification of the i-traits and growth-related traits

**Figure 5:**
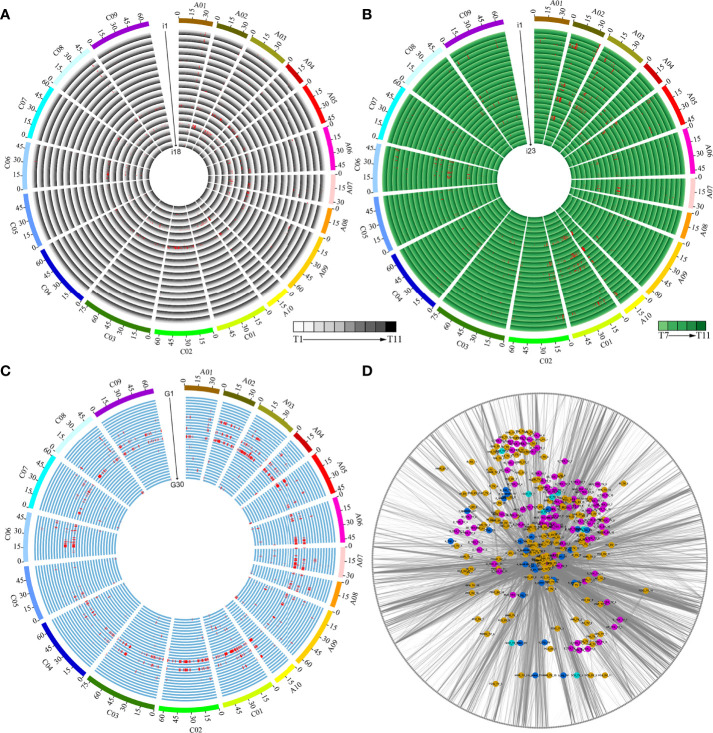
Dynamic QTLs detected in the population by GWAS. **(A)** QTLs responsible for the 18 i-traits in top view. i1-i18 represent GCV_TV, M_TEX_TV, SE_TEX_TV, S_TEX_TV, MU3_TEX_TV, U_TEX_TV, E_TEX_TV, TPA_TV, H_TV, W_TV, HWR_TV, FDNIC_TV, FDIC_TV, R_TV, PAR_TV, HA_TV, AC_TV, and GPA_TV, respectively. **(B)** QTLs responsible for the 23 i-traits in side view. i1-i23 represent M_TEX_SV, SE_TEX_SV, S_TEX_SV, MU3_TEX_SV, U_TEX_SV, E_TEX_SV, TPA_SV, H_SV, W_SV, HWR_SV, FDNIC_SV, FDIC_SV, R_SV, PAR_SV, HA_SV, AC_SV, GPA_SV, PC1_SV, PC2_SV, PC3_SV, PC4_SV, PC5_SV, and PC6_SV, respectively. The time points T1-T11 and T7-T11 are shown as circles with a colour gradient from light to dark, as indicated in the legend of **(A, B)**. **(C)** QTLs responsible for the 30 growth-related traits. G1-G30 represent a_linear_TV, b_linear_TV, a_power_TV, b_power_TV, a_Exp_TV, b_Exp_TV, a_log_TV, b_log_TV, a_quadratic_TV, b_quadratic_TV, c_quadratic_TV, a_sin_TV, b_sin_TV, c_sin_TV, d_sin_TV, a_linear_SV, b_linear_SV, a_power_SV, b_power_SV, a_Exp_SV, b_Exp_SV, a_log_SV, b_log_SV, a_quadratic_SV, b_quadratic_SV, c_”uadr’tic_SV, a_sin_SV, b_sin_SV, c_sin_SV, and d_sin_SV, respectively. **(D)** Network of associated bins with different traits. Green, blue, purple, and yellow nodes represent color traits, growth-related traits, histogram texture traits, and plant morphological traits (Details in **Supplementary Table S3**). The grey nodes of the outer ring represent the identified 602 nonredundant bins.

We performed GWAS using a set of 5,324,005 SNPs, which allowed us to identify the genetic basis of 41 dynamic i-traits and 30 growth-related traits. We detected a total of 4,088 loci associated with 66 traits across different time points, including 1,421, 1,753, and 914 loci associated with i-traits in top view, i-traits in side view, and growth-related traits, respectively (**Figures 5A**–**C**; **Supplementary Table S8**). The number of loci was significantly more than that identified for the same i-traits in the ISL population (Li et al., 2020), which suggested a higher detection power by combining high-throughput phenotyping and GWAS. Of which, 1,222 and 1,054 loci were responsible for the contributed i-traits in top view and side view, respectively. These associated loci were further involved in the 602 nonredundant QTLs, which was revealed by the trait-related association network (**Figure 5D**), suggesting the linkage or pleiotropy of locus. Of these, the largest proportion of nonredundant QTL were associated with a single trait (209, 34.7%), with those 105 nonredundant QTLs (17.4%) underlying single timepoint-dependent dynamic i-traits and 104 nonredundant QTLs (17.3%) underlying single growth-related traits. QTLs simultaneously affected the same trait at multiple growth stages, ranging from 2 to 7 and 2 to 5 for the dynamic i-traits in top view and side view, respectively (**Supplementary Figure S4**). The results reveal that these associated QTLs were expressed throughout multiple growth stages, which was almost impossible to detect by artificial phenotyping. For example, the QTLs involved in Bin161 on chromosome A07 simultaneously affected the PAR_TV at time points T4-T10 (**Supplementary Figure S5A**), which contributed to ecotype divergence and are associated with leaf angle and shape, petiole length, and plant compactness. And, the QTLs involved in Bin49 on chromosome A02 affected the MU3_TEX_SV at time points T7-T11 (**Supplementary Figure S5B**), which contributed to ecotype divergence and reflect the complex degree of leaf grayscale in vegetable state. Two i-traits in top view and all seven i-traits in side view were found to be associated with a number of loci at all eleven and five time points, respectively (**Figures 5A**, **B**; **Supplementary Table S8**). For the i-traits in top view and side view, the number of associated loci for each i-trait ranged from 1 to 218 (PAR_TV_5) at all 11 time points and from 1 to 134 (PC1_SV_7) at all 5 time points, with an average of 1.22-98.50 and 1.25-101.20 loci per time point, respectively. The QTLs were distributed nonrandomly throughout the rapeseed chromosome, with a maximum of 344 on C01 and a minimum of 102 on C5. However, there was the symmetrical distribution of QTL in A sub-genome (2,042) and C sub-genome (2,046) (*χ* ^2^-test, *p* = 0.95).

### Genetic divergence of the differentiated i-traits among the three ecotypes

**Figure 6:**
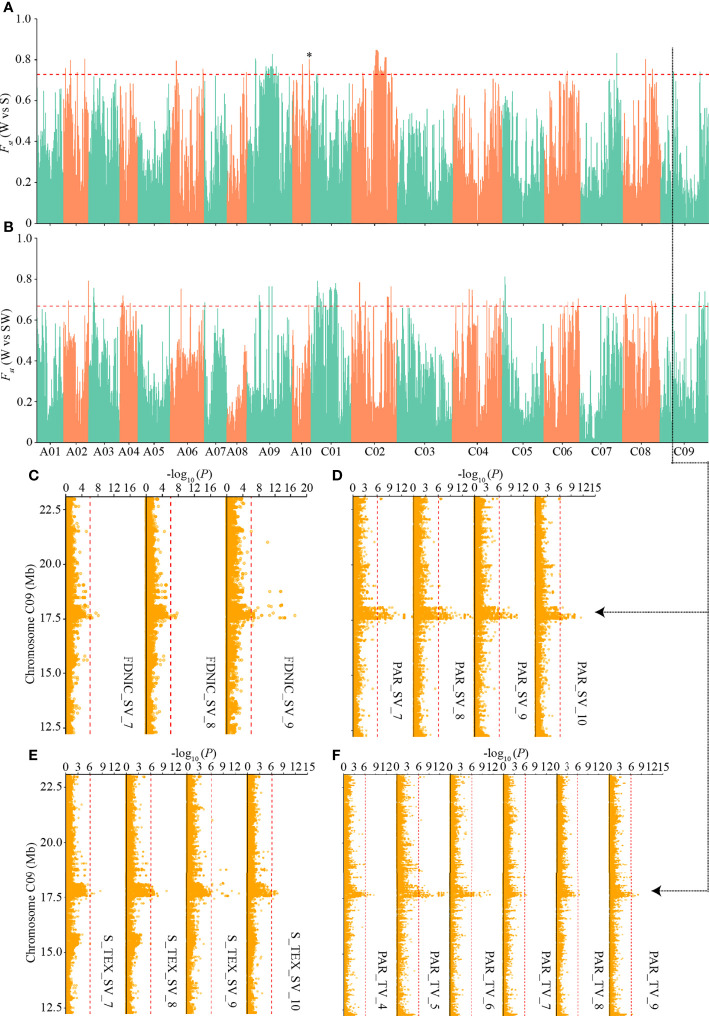
Genome-wide ecotype divergence with integration of multiple GWAS signals responsible for the differentiated i-traits. **(A, B)** Highly divergent regions between the winter and spring ecotypes **(A)** and winter and semi-winter ecotypes **(B)**. The horizontal red dashed lines indicate the thresholds (top 1% of *F_ST_* values). Region indicated by the asterisk was that surrounding *BnaA10.FLC*. **(C-F)** GWAS signals responsible for FDNIC_SV **(C)**, PAR_SV **(D)**, S_TEX_SV **(E)**, and PAR_TV **(F)** across multiple time points, which overlapped the common divergent regions on C09 chromosome between winter and spring/semi-winter ecotypes. The red vertical dashed lines in Manhattan plots indicate the threshold of GWAS (-log (*P* value) = 6.02).

To detect the genetic basis underlying the differentiated i-traits among the three ecotypes, we first calculated the pairwise population differentiation level and searched for genomic regions showing the highest level of fixation for SNPs (the top 1% of *F_ST_*) across different ecotypes. There were 78, 73, and 79 such regions with an *F_ST_* value greater than 0.72, 0.67, and 0.50 between W and S ecotypes, W and SW ecotypes, and S and SW ecotypes, respectively (**Supplementary Table S6**). These regions covered 9.11 Mb, 9,03 Mb, and 9.02 Mb in total, containing 605, 618, and 755 genes, respectively (**Supplementary Table S6**). We identified the local differentiation signals surrounding *BnaA10.FLC* on Chromosome A10 between W and S ecotype (**Figure 6A**), which had been detected previously and is a major association with seasonal crop type in rapeseed (Wu et al., 2019; Yin et al., 2020). This result prompted us to annotate the differentiation signals among the three ecotypes, in combination with the above-mentioned GWAS signals of differentiated i-traits.

Interestingly, when the physical QTLs of ecotype differentiated i-traits were compared with the 230 divergent regions, we found that 35, 33, and 42 divergent regions were located within known QTLs responsible for the differentiated i-traits between W and S, W and SW, and S and SW, respectively (**Supplementary Table S9**). Overall, the ratio of divergent regions overlapping with QTLs of ecotype differentiated i-traits (47.8%) was significantly more than that overlapping with QTLs of i-traits that little contributed to the divergence of ecotype (31.7%) (χ2-test, p = 2.66×10-4). For the divergent regions between W and S ecotype, a subset of 213 QTLs responsible for FDNIC_TV/SV, PAR_TV/SV, S_TEX _TV/SV, HA_TV, HWR_SV, SE_TEX_SV, and TPA_SV overlapped with the divergent regions. For the divergent regions between W and SW ecotype, a subset of 237 QTLs responsible for FDNIC_TV/SV, PAR_TV/SV, S_TEX _TV/SV, GCV_TV, HA_TV, M_TEX_TV, W_TV, HWR_SV, MU3_TEX_SV, SE_TEX_SV, and TPA_SV overlapped with the divergent regions. For the divergent regions between S and SW ecotype, a subset of 184 QTLs responsible for FDNIC_TV/SV, MU3_TEX_TV/SV, PAR_TV/SV, GCV_TV, M_TEX_TV, S_TEX _SV, SE_TEX_SV, and TPA_SV overlapped with the divergent regions. Of these, four common divergent regions between W and S/SW, located on chromosome A10, C01, and C09, were found to overlap with the dynamic QTLs responsible for FDNIC_SV, PAR_ SV, S_TEX _SV, and PAR_ TV at multiple growth stages (**Figure 6**; **Supplementary Figures S6**–**S8**). The strongest divergent regions between S and SW on chromosome C02 overlapped with the QTLs responsible for PAR_SV and FDNIC_TV at multiple growth stages (**Supplementary Figure S9**). Taken together, these results supported that alleles of i-traits contributing to ecotype divergence have experienced selection among the ecotype in rapeseed, which revealed that differentiation of i-traits among the ecotypes was contributed to by the genetic variations and differentiation.

## Data availability statement

The datasets presented in this study can be found in http://plantphenomics.hzau.edu.cn/usercrop/Rape/image/2014-2015-GWAS. The names of the repository/repositories and accession number(s) can be found in the article/**Supplementary Material**.

## Author contributions

HF, CG, HL, and WY performed the experiments, analyzed the data, and wrote the manuscript. ZL, GC, YG, QZ, ZG, and JW assisted in the experiment performance, data analysis, and database information construction. KL, HL, and WY supervised the project and designed the research. All authors contributed to the article and approved the submitted version.

## Funding

This work was supported by grants from the National Key Research and Development Program (2020YFD1000904-1-3), National Natural Science Foundation of China (32172028, U21A20205), Key projects of Natural Science Foundation of Hubei Province (2021CFA059), Fundamental Research Funds for the Central Universities (2021ZKPY006, 2662021JC008), and the HZAU-AGIS Cooperation Fund (SZYJY2022014).

## Conflict of interest

The authors declare that the research was conducted in the absence of any commercial or financial relationships that could be construed as a potential conflict of interest.

## Publisher’s note

All claims expressed in this article are solely those of the authors and do not necessarily represent those of their affiliated organizations, or those of the publisher, the editors and the reviewers. Any product that may be evaluated in this article, or claim that may be made by its manufacturer, is not guaranteed or endorsed by the publisher.
