# Supplementary material for: Machine learning assisted dynamic phenotypes and genomic variants help understand the ecotype divergence in rapeseed
Source: Front Plant Sci. 2022 Nov 15;13:1028779. doi: 10.3389/fpls.2022.1028779 (PMC9705987; doi:10.3389/fpls.2022.1028779)
Supplement: Supplementary file 1 [file DataSheet_1.pdf]

Supplementary Material

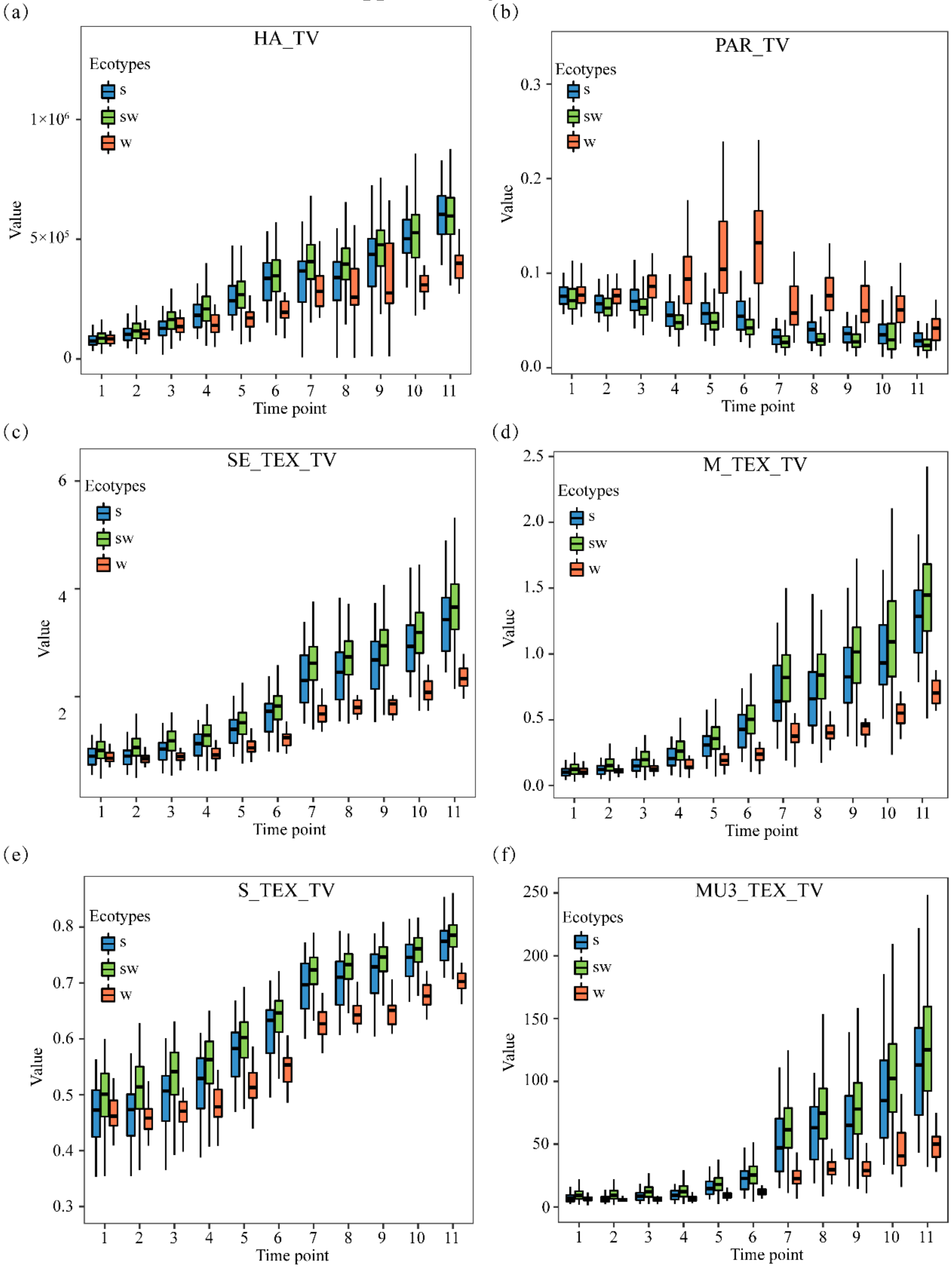

Supplementary Figure S1. Comparison of the contributed i-traits in top view among the three ecotypes. The box plots showing HA_TV (a), PAR_TV (b), SE_TEX_TV (c), M_TEX_TV (d), S_TEX_TV (e) and MU3_TEX_TV (f) from T1 to T11 in the three rapeseed ecotypes.

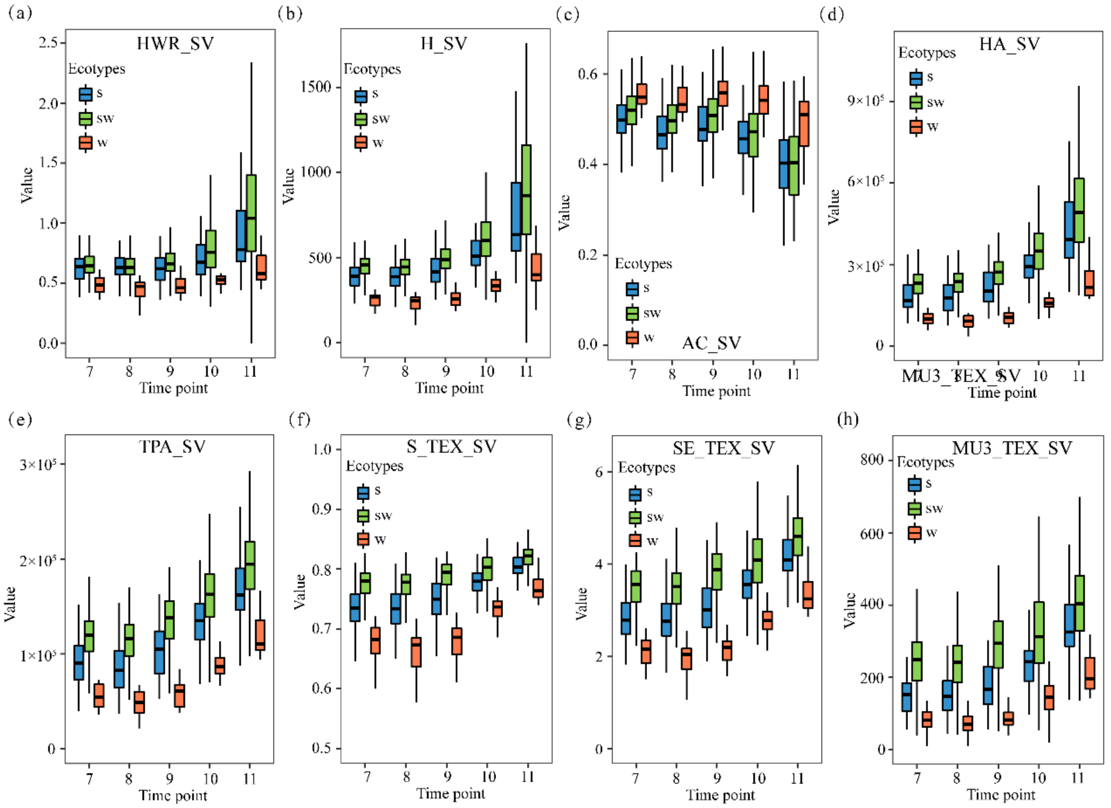

Supplementary Figure S2. Comparison of the contributed i-traits in side view among the three ecotypes. The box plots showing HWR_SV (a), H_SV (b), AC_SV (c), HA_SV (d), TPA_SV (e), S_TEX_SV (f), SE_TEX_SV (g) and MU3_TEX_SV (h) from T7 to T11 in the three rapeseed ecotypes.

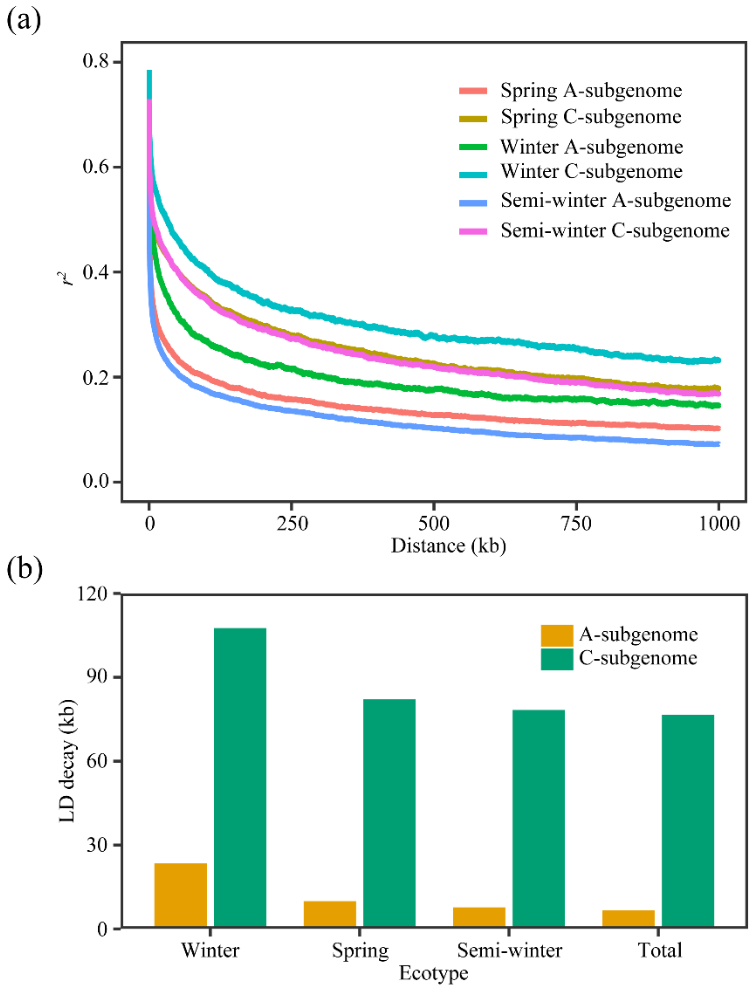

Supplementary Figure S3. LD in the A and C subgenomes among the three ecotypes. (a) Diagram showing the decay of LD in the A and C subgenomes among the three ecotypes. (b) Decay distance of LD (1/2 max r^2) in the A and C subgenomes among the three ecotypes.

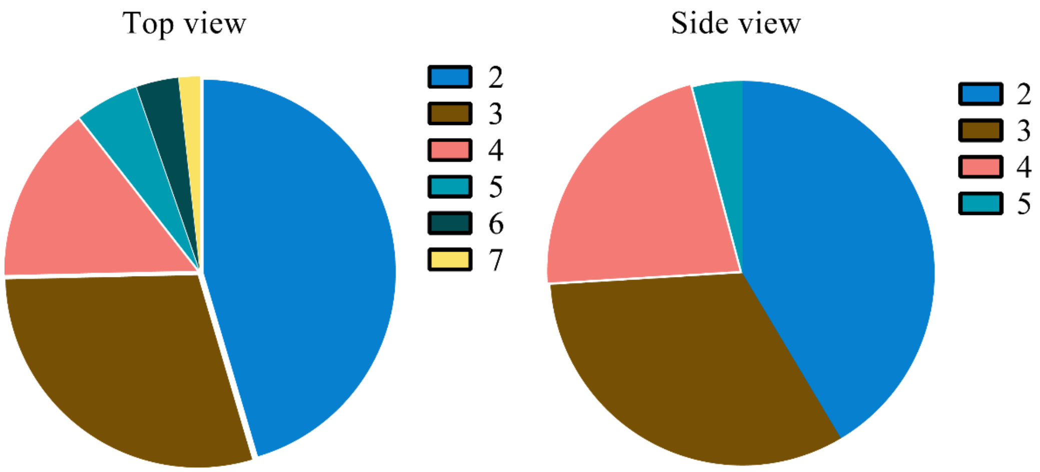

Supplementary Figure S4. Distribution of QTLs responsible for the same traits across multiple stages in top view (a) and side view (b).

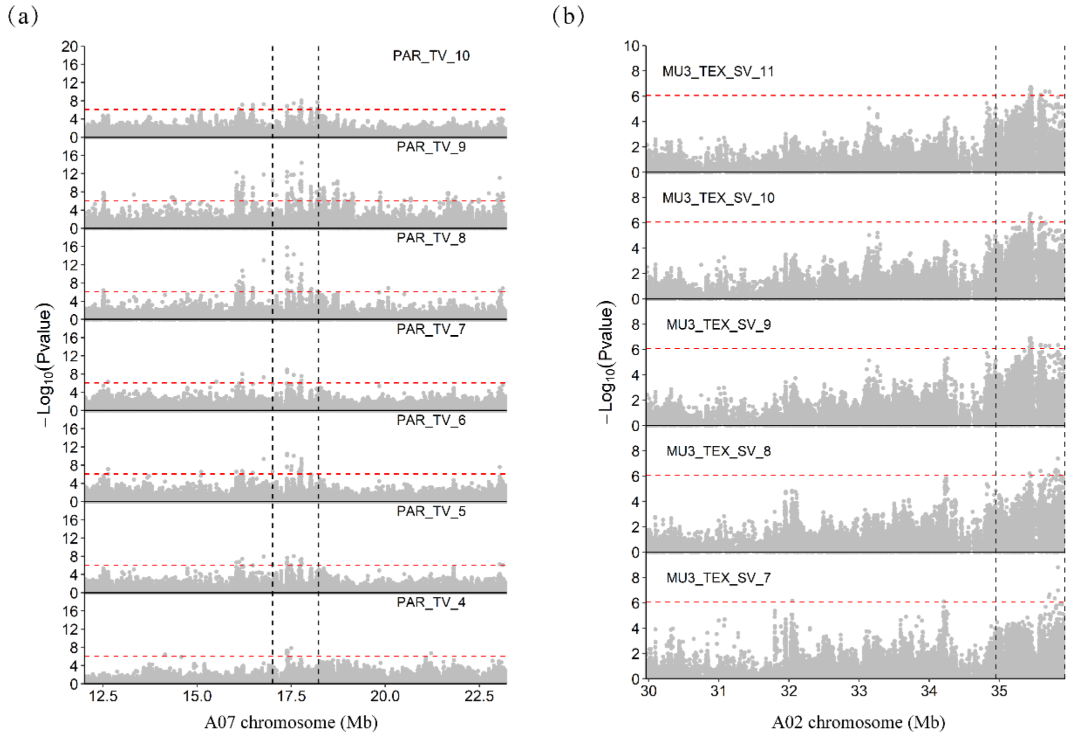

Supplementary Figure S5. Dynamic QTLs responsible for the PAR_TV (a) and MU3_TEX_SV (b) across multiple stages. The red horizontal dashed lines in Manhattan plots indicate the threshold of GWAS (-log (*P* value) = 6.02).

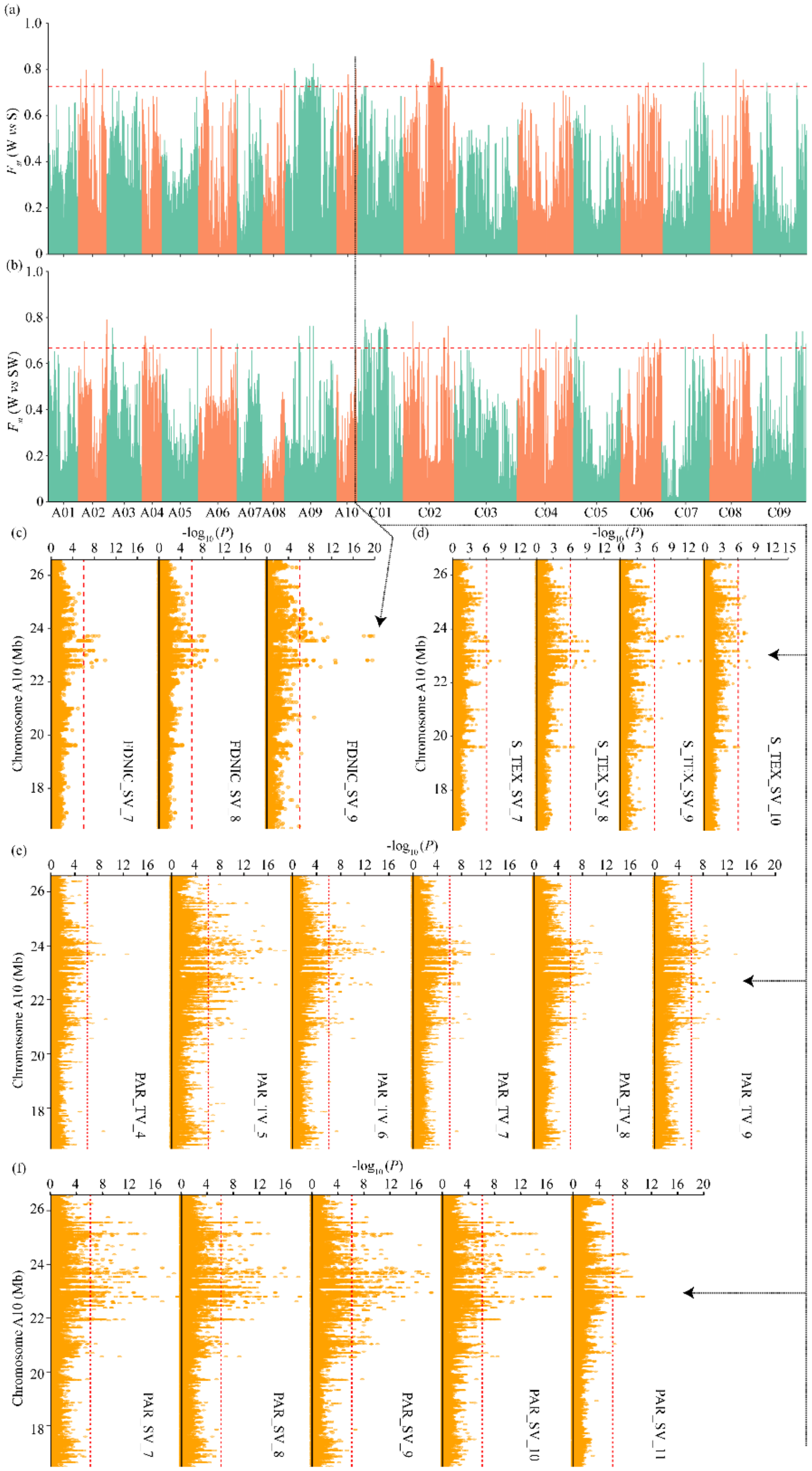

Supplementary Figure S6. GWAS signals that overlapped with common divergent regions on chromosome A10 between winter and spring/semi-winter ecotype. (a, b) Highly divergent regions between the winter and spring ecotypes (a), winter and semi-winter ecotypes (b). The horizontal red dashed lines indicate the thresholds (top 1% of F_{ST} values). (c-f) GWAS signals responsible for FDNIC_SV (c), S_TEX_SV (d), PAR_TV (e) and PAR_SV (f) across multiple time points. The red vertical dashed lines in Manhattan plots indicate the threshold of GWAS (-log (*P* value) = 6.02).

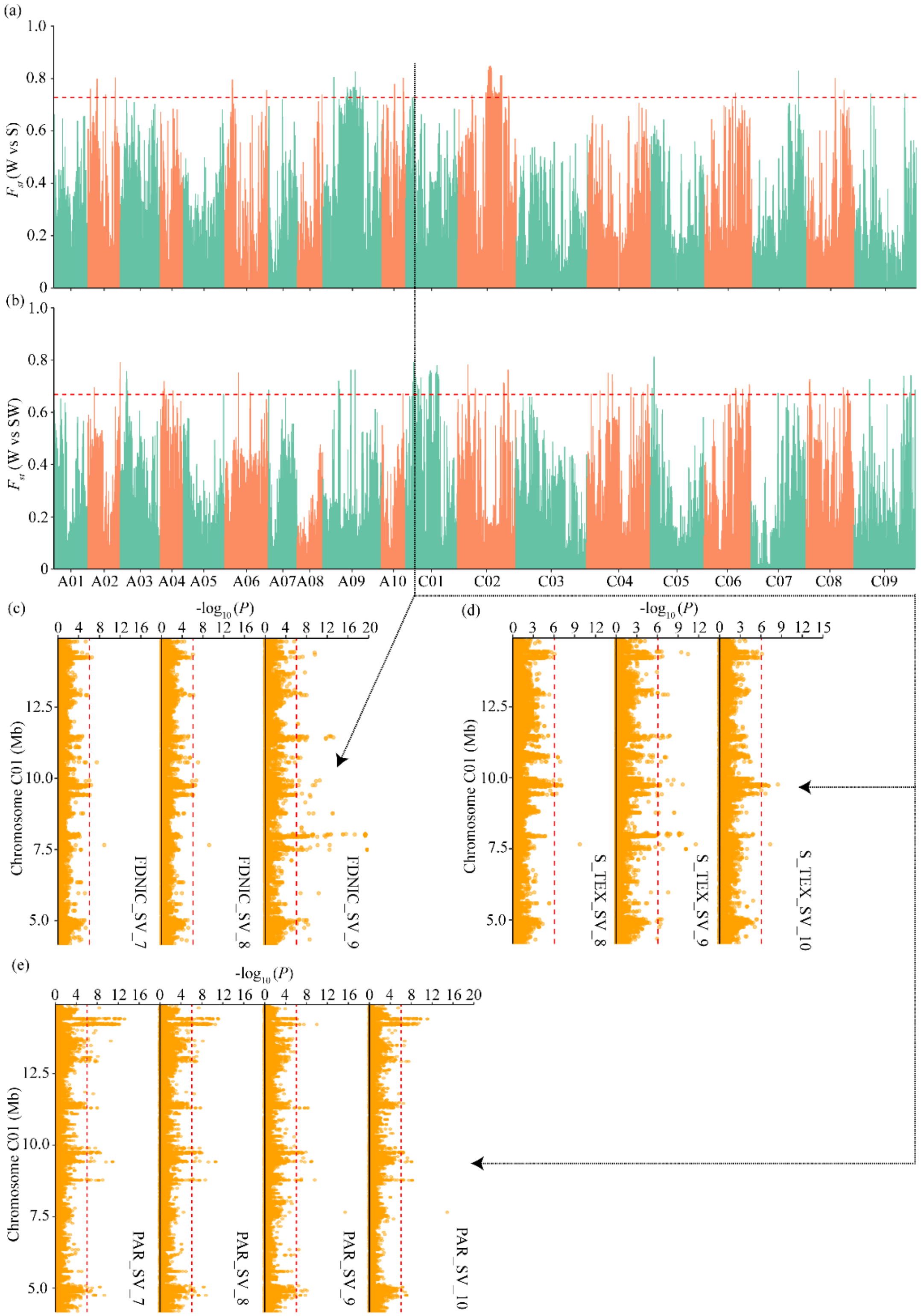

Supplementary Figure S7. GWAS signals that overlapped with common divergent regions on chromosome C01 between winter and spring/semi-winter ecotype. (a, b) Highly divergent regions between the winter and spring ecotypes (a), winter and semi-winter ecotypes (b). The horizontal red dashed lines indicate the thresholds (top 1% of F_{ST} values). (c-e) GWAS signals responsible for FDNIC_SV (c), S_TEX_SV (d) and PAR_SV (e) across multiple time points. The red vertical dashed lines in Manhattan plots indicate the threshold of GWAS (-log (*P* value) = 6.02).

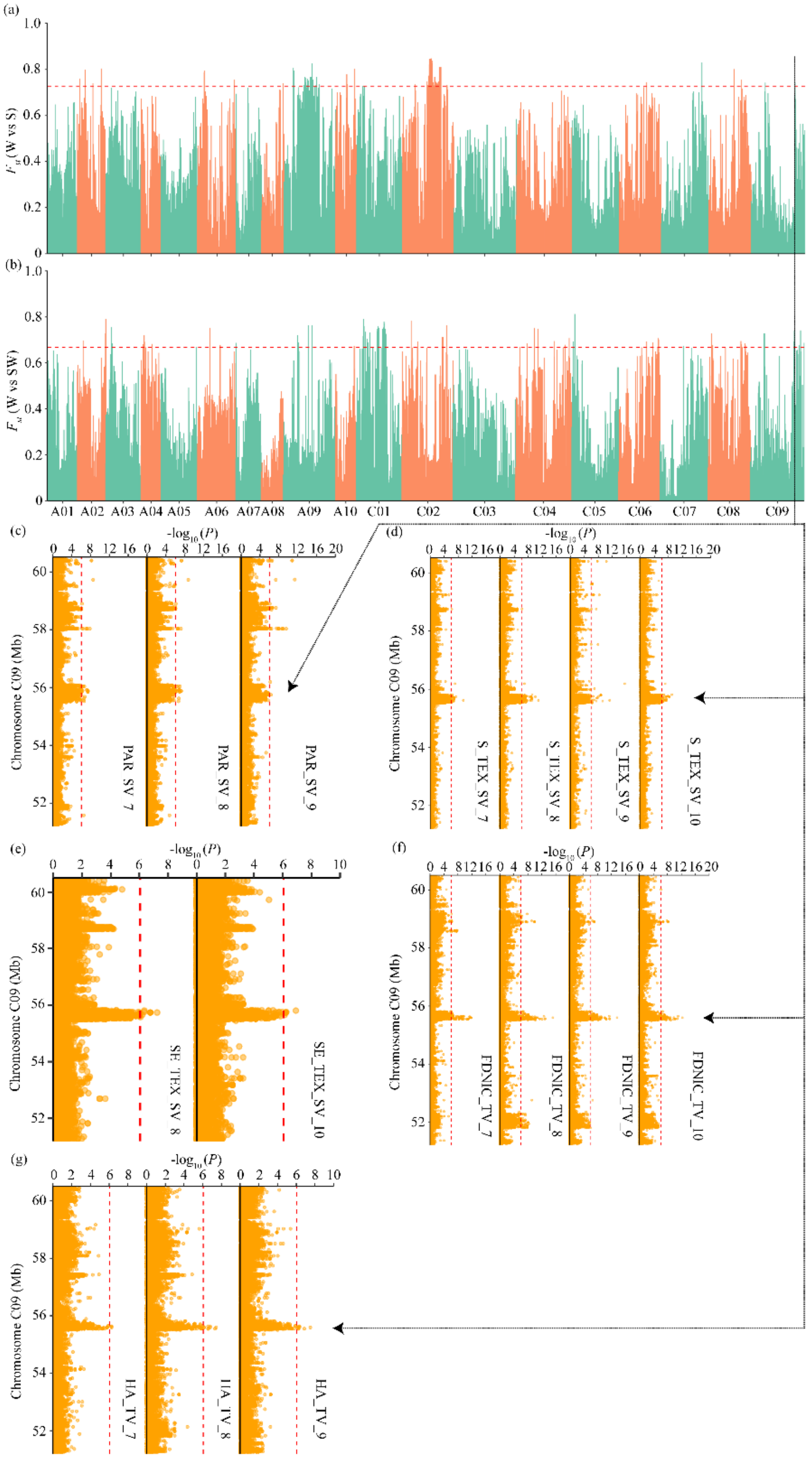

Supplementary Figure S8. GWAS signals that overlapped with common divergent regions on chromosome C09 between winter and spring/semi-winter ecotype. (a, b) Highly divergent regions between the winter and spring ecotypes (a), winter and semi-winter ecotypes (b). The horizontal red dashed lines indicate the thresholds (top 1% of F_{ST} values). (c-g) GWAS signals responsible for PAR_SV (c), S_TEX_SV (d), SE_TEX_SV (e), FDNIC_TV (f) and HA_TV (g) across multiple time points. The red vertical dashed lines in Manhattan plots indicate the threshold of GWAS (-log (*P* value) = 6.02)

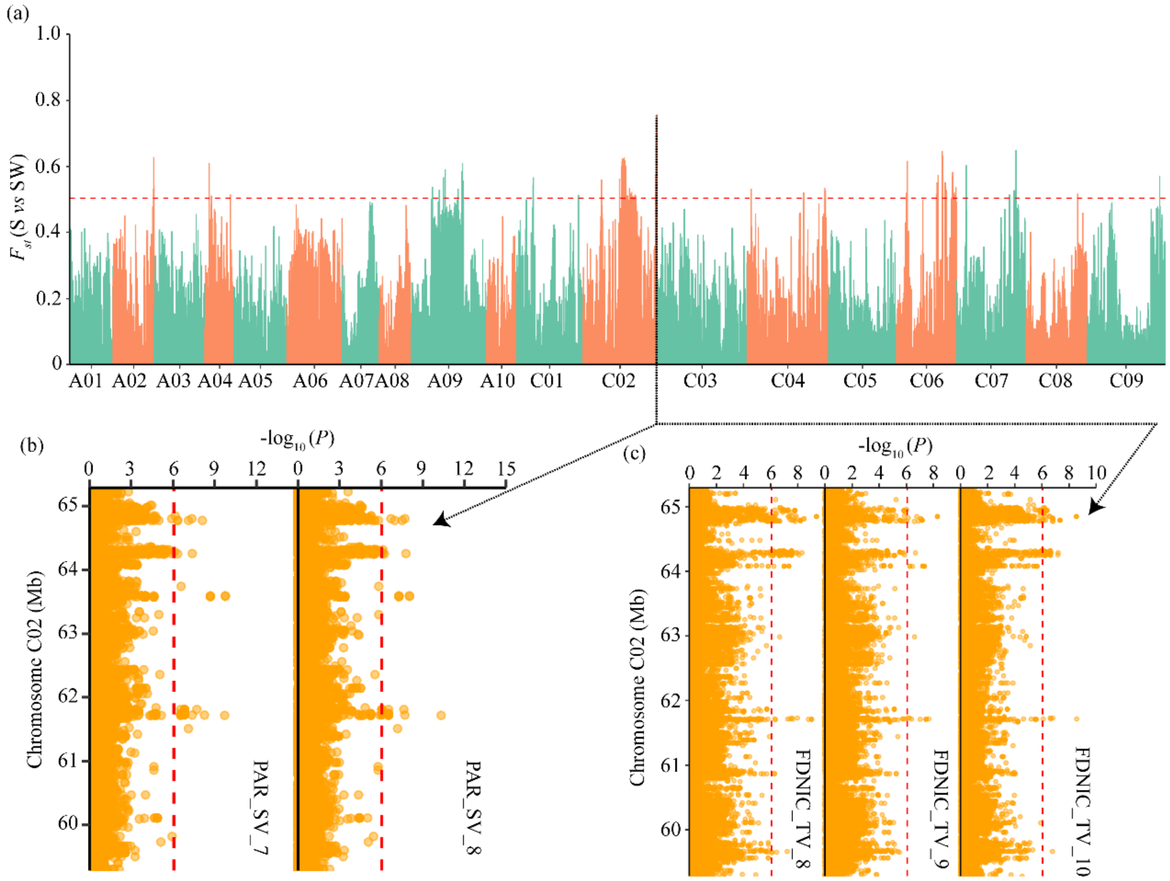

Supplementary Figure S9. GWAS signals that overlapped with the highest divergent regions on chromosome C02 between spring and semi-winter ecotype. (a) Highly divergent regions between the spring and semi-winter ecotypes. The horizontal red dashed lines indicate the thresholds (top 1% of F_{ST} values). (b and c) GWAS signals responsible for PAR_SV (b) and FDNIC_TV (c) across multiple time points. The red vertical dashed lines in Manhattan plots indicate the threshold of GWAS (-log (*P* value) = 6.02).
