# Supplementary material for: Machine learning assisted dynamic phenotypes and genomic variants help understand the ecotype divergence in rapeseed
Source: Front Plant Sci. 2022 Nov 15;13:1028779. doi: 10.3389/fpls.2022.1028779 (PMC9705987; doi:10.3389/fpls.2022.1028779)
Supplement: Supplementary file 2 [file DataSheet_2.docx]

Supplementary Material

**Supplementary Table S2. Inspection dates with temperature condition during the growing season.**

| **Time points** | **Date** | **Type of i-trait** | **Temperature (℃)** | |
| --- | --- | --- | --- | --- |
|  |  |  | Min | Max |
| T1 | 2014.12.16 | Top view | 0 | 8 |
| T2 | 2014.12.23 | Top view | -2 | 12 |
| T3 | 2014.12.30 | Top view | 1 | 17 |
| T4 | 2015.1.6 | Top view | 3 | 6 |
| T5 | 2015.1.13 | Top view | 4 | 12 |
| T6 | 2015.1.20 | Top view | 5 | 13 |
| T7 | 2015.1.27 | Top and side view | 3 | 5 |
| T8 | 2015.2.3 | Top and side view | -2 | 8 |
| T9 | 2015.2.10 | Top and side view | -3 | 12 |
| T10 | 2015.2.17 | Top and side view | 4 | 19 |
| T11 | 2015.2.24 | Top and side view | 2 | 14 |

**Supplementary Table S5 List of i-traits contributing to the divergence of ecotype.**

| **Direction** | **i-traits** |
| --- | --- |
| Top view | MU3_TEX_TV, SE_TEX_TV, S_TEX_TV, M_TEX_TV, HA_TV, FDNIC_TV, GCV_TV, PAR_TV, W_TV |
| Side view | SE_TEX_SV, S_TEX_SV, MU3_TEX_SV, TPA_SV, H_SV,  HWR_SV, FDNIC_SV, PAR_SV, HA_SV, AC_SV |

**Supplementary Table S6. Summary of genetic variation across the three ecotypes in rapeseed.**

|  |  | all | Ecotype | | |
| --- | --- | --- | --- | --- | --- |
|  |  |  | Winter | Spring | semi-winter |
| No. of accessions |  | 171 | 14 | 24 | 133 |
| variation | SNPs | 5,324,005 | 3,590,454 | 4,775,778 | 5,323,607 |
|  | π (10^-3^) | 1.78 | 1.17 | 1.56 | 1.68 |
| LD | r^2^= 1/2 max r^2^ | 0.33 | 0.39 | 0.35 | 0.34 |
|  | LD decay（Kb, *r*^2^= 1/2 max *r*^2^） | 20.6 | 52 | 23.5 | 18.4 |
|  |  |  | Spring *vs.* Winter | Winter *vs* Semi-Winter | Spring *vs.* Semi-Winter |
| Ecotype divergence | *Fst*, top 1% threshold |  | 0.72 | 0.67 | 0.50 |
|  | Regions |  | 78 | 73 | 79 |
|  | Length of Regions (Mb) |  | 9.11 | 9.03 | 9.02 |
|  | Genes |  | 605 | 618 | 755 |

**Supplementary Table S7. Distribution and annotation of SNPs across the genome.**

| **Chr** | **SNP No.** | **Intergenic** | **CDS** | **Intron** | **Synonymous SNP** | **Nonsynonymous SNP** |
| --- | --- | --- | --- | --- | --- | --- |
| A01 | 246,045 | 98,526 | 48,390 | 40,677 | 31,140 | 17,036 |
| A02 | 280,378 | 121,006 | 47,676 | 43,153 | 29,508 | 17,908 |
| A03 | 340,257 | 111,138 | 73,905 | 64,002 | 47,235 | 26,333 |
| A04 | 227,537 | 100,388 | 39,484 | 33,540 | 24,601 | 14,678 |
| A05 | 287,527 | 131,716 | 50,698 | 41,222 | 32,929 | 17,529 |
| A06 | 269,187 | 109,573 | 51,829 | 44,184 | 32,988 | 18,609 |
| A07 | 271,083 | 110,763 | 49,830 | 42,128 | 31,954 | 17,657 |
| A08 | 184,241 | 73,870 | 34,871 | 30,426 | 21,753 | 12,940 |
| A09 | 385,840 | 164,761 | 68,038 | 59,872 | 43,273 | 24,410 |
| A10 | 199,939 | 72,582 | 43,060 | 36,687 | 27,710 | 15,184 |
| C01 | 322,489 | 194,261 | 35,201 | 35,989 | 19,793 | 15,101 |
| C02 | 364,218 | 224,394 | 35,354 | 41,812 | 18,972 | 15,945 |
| C03 | 418,344 | 209,128 | 54,288 | 58,943 | 28,996 | 24,834 |
| C04 | 355,925 | 218,050 | 34,645 | 39,095 | 18,570 | 15,709 |
| C05 | 182,882 | 94,720 | 25,398 | 25,656 | 14,035 | 11,176 |
| C06 | 253,228 | 148,041 | 27,080 | 29,652 | 14,239 | 12,549 |
| C07 | 282,921 | 165,307 | 30,152 | 33,651 | 16,084 | 13,814 |
| C08 | 220,055 | 120,727 | 25,371 | 28,056 | 13,861 | 11,300 |
| C09 | 231,909 | 120,309 | 32,690 | 31,901 | 18,527 | 13,900 |
